# Molecular Dynamics Simulation Analysis of Damage and Expansion Process of Nanoindentation Single-Crystal 3C-SiC Carbide Specimens at Different Temperature

**DOI:** 10.3390/nano13020235

**Published:** 2023-01-04

**Authors:** Xiang Ning, Nanxing Wu, Mengjuan Zhong, Yuwei Wen, Bin Li, Yi Jiang

**Affiliations:** 1 School of Mechanical and Electronic Engineering, Jingdezhen Ceramic University, Jingdezhen 333403, China; 2 Laboratory of Ceramic Material Processing Technology Engineering, Jingdezhen 333403, China

**Keywords:** 3C-SiC, nanoindentation, temperature, stress strain, dislocation evolution

## Abstract

The molecular dynamics method was used to analyze the influence of simulated temperature on the damage expansion process of the 3C-SiC sample under nano-indentation loading in order to study the influence of temperature on the internal damage and expansion mechanism of the 3C-SiC single crystal sample further during the nano-indentation loading process. A simulation test platform for diamond indenter indentation was established. The process of stress and strain distribution, dislocation evolution, dislocation expansion and potential energy change were analyzed, combined with the radial distribution function and load displacement curve. The influence of temperature on the 3C-SiC material was discussed. The variation trend of the potential energy-step curve is basically the same at the temperatures of 0 K, 300 K, 600 K and 900 K. The difference in strain distribution was characterized by the influence of temperature on stress intensity, expansion direction and type. The microcosmic manifestation is the significant difference in the dislocation slip at low temperature. In the process of dislocation evolution and expansion, dislocation climbs at room temperature and increases at high temperature, which is closely related to energy release. This study has certain guiding significance for investigating the internal damage difference and temperature effect of the 3C-SiC sample.

## 1. Introduction

3C-SiC is widely used in electronic equipment manufacturing, graphene preparation [1], aerospace equipment [2,3], nuclear industry and other high-end equipment because of its high hard-corrosion resistance, radiation resistance [4,5] and excellent electrical properties. The properties of 3C-SiC materials are greatly affected by temperature. It tends to affect the accuracy and durability of equipment applications during application and processing. However, the traditional material detection technology has some problems, such as low precision and difficult monitoring. The emerging nanoindentation technology [6,7] is a good solution to the current problem. Continuous changes in load are controlled by a computer program. This method has the advantages of real-time and high precision, and can monitor the load-displacement curve, stress-strain, dislocation behavior and other nanoscale mechanical properties. Based on molecular dynamics [8], simulation temperature on the influence of nanoindentation loading 3C-SiC damage and expansion process, which has important research significance for the setting of actual processing temperature conditions, was assessed through the analysis and evaluation of application performance.

At present, scholars at home and abroad conducted a series of studies on the influence of temperature on the microscopic properties of nanoindentation process materials by molecular dynamics methods. Huang et al. [9] studied the influence of temperature on the process of single crystal copper nanoindentation based on molecular dynamics and found that, with the increase in temperature, the area of deformation in contact with the indenter expanded; the degree of plastic deformation weakened; and the potential energy value of the system was decreasing while maintaining the basic indifference in the change trend at various temperatures. Sun et al. [10,11] used molecular dynamics simulation to analyze the dislocation generation, load displacement curve and stress strain of 3C-SiC specimens under nanoindentation, etc.; they also proved that there was a reversible amorphous quasi-elastic deformation induced by reversible amorphism after pure elastic deformation but before plastic deformation, the elastoplastic deformation mechanism of 3C-SiC under the action of spherical indenter nanoindentation, and proposed the “lasso” like mechanism and extended “lasso” on the formation mechanism of prismatic loops in crystals. Two theoretical guidelines for the class mechanism explain the evolution mechanism of 3C-SiC plastic deformation. Elchin M et al. [12,13] studied the change in the surface of 3C-SiC particles with temperature and the heating rate, determined the specific heat capacity and Gibbs free energy at different heating rates, which has important reference significance for the field of 3C-SiC thermal processing, and studied the nanoindentation of Cu/Au thin films at different temperatures by the deposition method and ideal modeling method based on lattice constant, then visualized and analyzed the lattice structure based on open software Ovito. It was found that the hardness of the film weakened with the increase in temperature. Marinova et al. [14,15] discussed the effect of the temperature gradient on the structural quality of each layer of 3C-SiC layers’ growth, studied the effect of temperature on structure, phase evolution and mechanical properties, and determined that the aspect ratio of 3C-SiC is maintained well below 1900 °C. The study of the mechanism has the influence of temperature factors on the mechanical behavior, damage and expansion of materials and has received extensive attention from scholars at home and abroad, but the study of the evolution mechanism of micromechanical properties under the variable temperature conditions of 3C-SiC specimens is particularly thin. In order to fill the gap in the influence of temperature factors on the micromechanical properties of the 3C-SiC specimen processing process, molecular dynamics methods were used to study the influence of temperature on the damage and expansion process of nanoindentation 3C-SiC.

In this paper, the molecular dynamics analysis method was used to simulate the change state of 3C-SiC nanoindentation at low temperature, room temperature, medium-high temperature, moderate temperature and high temperature. We analyzed the radial distribution curves, stress-strain, dislocation behavior and potential energy variation curves at different temperatures, and deeply analyzed the microscopic deformation, damage and expansion behaviors of 3C-SiC samples under the influence of temperature changes in the process of nanoindentation loading. This study has a certain guiding significance for exploring the damage and elongation mechanism of 3C-SiC in different environments.

## 2. Model Building

### 2.1. Molecular Dynamics Model of Single Crystal 3C-SiC Indentation Process

**Figure 1:**
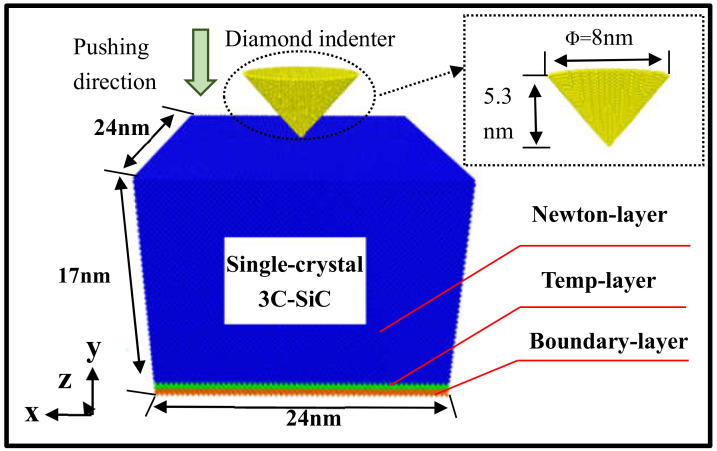
Nanoindentation single crystal 3C-SiC model diagram.

The establishment of a reasonable simulation model is a prerequisite for ensuring the accuracy of the experimental simulation process of single crystal 3C-SiC nanoindentation. As shown in Figure 1, the three-dimensional molecular dynamics model of a single crystal 3C-SiC nanoindentation consists of a Rockwell diamond indenter and a single crystal 3C-SiC matrix.

In the simulation, a simulation box of 24 nm × 27.5 nm × 24 nm is established as the boundary, involving a total of 1,039,657 atoms. The yellow conical part is a Rockwell diamond indenter with a diameter of 8 nm and a height of 5.3 nm; the diamond hardness and elastic modulus are very high, which are set as an absolute rigid body, containing 24,409 atoms. The matrix size of the 3C-SiC test to be pressed is 24 nm × 17 nm × 24 nm, and, in order to maintain the balance and stability of the physical model, the 3C-SiC matrix is finely divided into three regions, namely the brown boundary area, the green constant temperature region and the blue Newtonian layer area. The atoms of the boundary layer are set at a steady state to prevent the substrate from slipping during the indentation process; the atoms of the thermostatic layer play a role in buffering, maintaining temperature stability, and the atoms of the Newtonian layer are the experimental research area. The atoms of the thermostat layer and the Newtonian layer both conform to Newton’s second law of motion, which participate in the calculation of the potential energy and mechanics of the atoms in the whole process; the three regions involve a total of 1,015,040 atoms. In order to study the influence of temperature on the damage and expansion process of nanoindentation single crystal silicon carbide, the initial temperature of the system was set to 0 K, 300 K, 600 K, 900 K; the low-temperature, room temperature, medium-high temperature and high-temperature environments were mapped, respectively. In order to maintain the temperature stability of the thermosphere atoms, adjust the velocity of the thermosphere atoms every 100 steps and, in order to eliminate the “size effect” [16] and “surface effect” in the nanoscale [17], set the X and Z directions of the simulation box as periodic boundaries and the Y direction as free boundaries to ensure that the calculation results are accurate. The calculation parameters simulated in this paper are shown in Table 1.

### 2.2. Interatomic Potential Function

The interactions between the atoms of materials fundamentally determine the macroscopic fundamental properties of materials. The interaction between atoms is characterized by the potential function, which is closely related to the elastic constant, binding energy and hardness of materials. Choosing the right potential function is a prerequisite for correct calculation. In the simulation process, the motion states of each atom are calculated accurately so as to obtain the various state quantities and thermodynamic properties of the system. 3C-SiC is a fcc crystal; the lattice constant a = 4.259 Å; and the Tersoff potential function [18] based on the proximity interaction between atoms can accurately describe the potential function of the empirical bond sequence potential of the semiconductor atom interaction such as CN and SiN in the early stage of indentation. When the indentation simulation is carried out until the middle of relaxation, a large number of covalent bonds are broken; the force and velocity of the atom are out of the actual situation of the Tersoff potential function constraint. The Vashishta potential function [19] composed of a two-body potential, three-body potential superposition overlaps between C and Si atoms during the simulated relaxation process under the condition of the shrinkage periodic boundary; in order to calculate the position and velocity of the atoms at each moment in the indentation loading simulation process accurately by computer, this paper simulates the combined potential of the Tersoff potential function and the Vashishta potential function to describe the role between C-C, C-Si and Si-Si atoms accurately. It takes the following forms:

3C-SiC atom-to-atom interaction energy:

(1) $$\varphi \left(d\right)=E_{b\sigma }+E_{b\pi }+\sqrt[{12}]{C\left(\frac{a_{0}}{d}\right)}$$

Among them, *E_bσ_* is the polarization energy of the interatomic *σ* bond; *E_bπ_* is the polarization energy of the interatomic *π* bond; *C* is the constant coefficient, which is related to the bond energy parameter; *d* is the bond length, and *a*_0_ is the valence parameter of the *σ* bond and *π* bond.

The expression for potential energy between atoms is

(2) $$E=\frac{1}{2}\underset{i}{\sum }\underset{i\neq j}{\sum }U_{ij}$$

Tersoff potential function:

(3) $$U_{ij}=f_{c}\left(\delta r_{ij}\right)\left[f_{r}\left(\delta r_{ij}\right)+b_{ij}f_{a}\left(\delta r_{ij}\right)\right]$$

Vashishta potential function:

(4) $$V=\underset{i<j}{\sum }V_{ij}^{A}\left(r_{ij}\right)+\underset{i+j<k}{\sum }V_{ijk}^{B}\left(r_{ij},r_{ik}\right)$$

Among them, *U_ij_* is the total potential energy; *f_a_* is the interatomic electrostatic force function, which is related to the covalent bond energy; *f_r_* is the interatomic electromagnetic repulsion function, which is related to the quadrature of electron fluctuations; *f_c_* represents the truncation function of the interaction between atoms; *δr_ij_* represents the truncation radius between *i* atoms and *j* atoms; and *b_ij_* represents the multibody action function related to the bond angle. *V* represents the total energy; *V_ij_* represents the two-body potential energy; *V_ijk_* represents the three-body potential energy.

## 3. Numerical Solution Process

For the molecular dynamics simulation calculation to determine the motion of each atom in the model at this moment and the next moment, it is necessary to set a reasonable simulation environment to ensure the accuracy of the calculation results and the feasibility of the simulation. In this simulation, in order to ensure that the temperature of the system remains constant during relaxation, the isothermal isobaric ensemble (NPT) is set as the initial condition for the simulation. In the process of indentation simulation, the diamond indenter is pressed down in the negative direction of the Y axis, and its initial speed is set to 20 m/s; in the isotherm-isopressure system environment, it is necessary to meet the conservation law of energy in the classical force field to maintain the system balance, and set the atomic speed to adjust in real time to ensure that the sum of the kinetic energy and potential energy of the system in the whole process of simulation is constant. In order to fully simulate the loading process of nanoindentation in 0 K, 300 K, 600 K and 900 K environments, ensuring the accuracy of the calculation results, the total relaxation is set to 1000 fs, and the indentation depth is 10 nm.

In the process of molecular dynamics simulation, the microscopic motion of particles in the molecular force field conforms to Newton’s second law of motion, and the Velocity-Verlet [20] algorithm proposed by Swope is used to solve the displacement, velocity and acceleration of the particles that can be accurately obtained with the specific solution process as follows:

Velocity-Verlet numerical solution algorithm

(5) $$\frac{d^{2}r_{i}}{dt^{2}}=\frac{1}{m_{i}}\sum_{i\neq j}F_{i}\left(r_{ij}\right)\;\left(i=1,2,\ldots ,N\right)$$

(6) $$r_{i}\left(t+\delta t\right)-r_{i}\left(t\right)+v_{i}\left(t\right)\delta t+\frac{1}{2}a_{i}\left(t\right)\delta t^{2}$$

(7) $$v_{i}\left(t+\delta t\right)=v_{i}\left(t\right)+\frac{1}{2}\left[a_{i}\left(t+\delta t\right)+a_{i}\left(t\right)\right]\delta t$$

The Debye temperature conversion formula for 3C-SiC is:

(8) $$\Theta \left(K\right)=\frac{h}{k}\sqrt[{3}]{\left|6\pi^{2}K^{\frac{1}{2}}n\right|}f\left(k\right)\sqrt{\frac{Bs}{M}}$$

where Θ (*K*) is the Debye temperature; *m* is the mass of the nth atom; *n* is the number of atoms in the crystal’s protocells; *k* is the Boltzmann constant; *k* is the Poisson ratio; *B_S_* is the elastic modulus of the adiabatic body; and *V* is the crystal volume.

## 4. Results and Analysis

### 4.1. Single Crystal 3C-SiC Temperature Effect Analysis

By calculating the local spatial distribution, representing the aggregation state of particles, studying the correlation between the ordering of matter and the interaction between atoms, analyzing the radial distribution function to study the temperature effect of single crystal 3C-SiC [21], the formula is as follows:

(9) $$\rho g\left(r\right)=\frac{1}{N}\left(\overset{n}{\underset{i=1}{\sum }}\delta \left(r+\Delta R_{ij}\right)\right)$$

where *g*(*r*) is the chance of finding an atom in the range *r*~*r* + Δ*r*; *ρ* is the average density of the system; Δ*R_ij_* represents the distance between *i* and *j* atoms; *δ* is the Dirac symbol; and *N* is the number of atoms.

**Figure 2:**
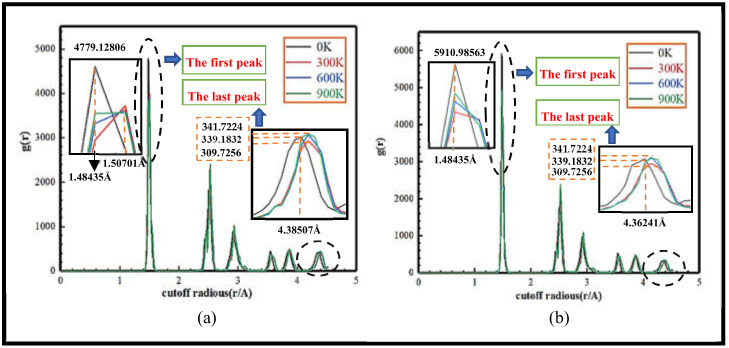
Radial distribution curve at different temperatures. (**a**) Radial distribution function of 2 nm, (**b**) Radial distribution function of 10 nm.

Regarding 3C-SiC in (010) crystal plane different temperatures between the C-Si atom radial distribution function, as shown in Figure 2, which can be seen from Figure 2, temperature on the radial distribution function of the overall influence is not very large; the aggregation state region of the atom is more concentrated. In Figure 2a, when the indentation depth is 2 nm, the first and last peaks are reached at the truncation radius r = 1.48435 A and r = 4.38507 A, respectively. The maximum value of g(r) is reached at the low temperature of 0 K, corresponding to 4779.12806 and 339.1832. The correlations between particles from strong to weak are 900 K, 600 K and 300 K. As shown in Figure 2b, the indentation depth is 10 nm; the first and last peaks are first reached at the truncation radius r = 1.48435 A and r = 4.36241 A, respectively, which is the same as the law when the indentation depth is 2 nm, and the two peaks are the largest at 0 K low temperature g(r), corresponding to the peaks of 5910.98563 and 339.1832, and the correlation between particles is 900 K > 600 K > 300 K. The radial distribution function under the depth of 2 nm and 10 nm indentation was showing a downward trend, and the probability of atomic distribution at the truncation radius of 1.48435 A is the highest, while the corresponding peak g(r) of 10 nm is significantly greater than the corresponding peak of 2 nm, and the truncation radius of 10 nm to reach the last peak is slightly less than the truncation radius of 2 nm to reach the last peak, but the difference between the corresponding peaks at the two indentations is relatively small, which indicates that the depth of the indentation increases. The covalent bond between atoms around the contact area with the indenter is fractured and reconstructed jointly, and the motion of the atomic sequence changes. In summary, the influence of temperature on the radial distribution function of the 3C-SiC nanoindentation is not significant in general, and the change trend in the radial distribution function of different indentation depths is basically the same under the conditions of low temperature, room temperature, medium-high temperature and high temperature, and the radial distribution relationship of atoms under temperature conditions of 900 K and 600 K fluctuates between 0 K and 300 K temperatures, and the relationship between high temperature, medium and high temperature and radial distribution curves at room temperature coincides in general. In the 0 K low temperature environment, the motion between particles is gentle, and the covalent bond between atoms exists stably and acts steadily, which can maintain the close connection state of the atoms and make the C-Si atoms show a high aggregation state. The temperature increase from 0 K to 900 K has little effect on 3C-SiC materials, and to a certain extent, it is verified that 3C-Si materials have high-temperature resistance characteristics.

### 4.2. Damage Analysis of Temperature Influence Indentation Loading Process

**Figure 3:**
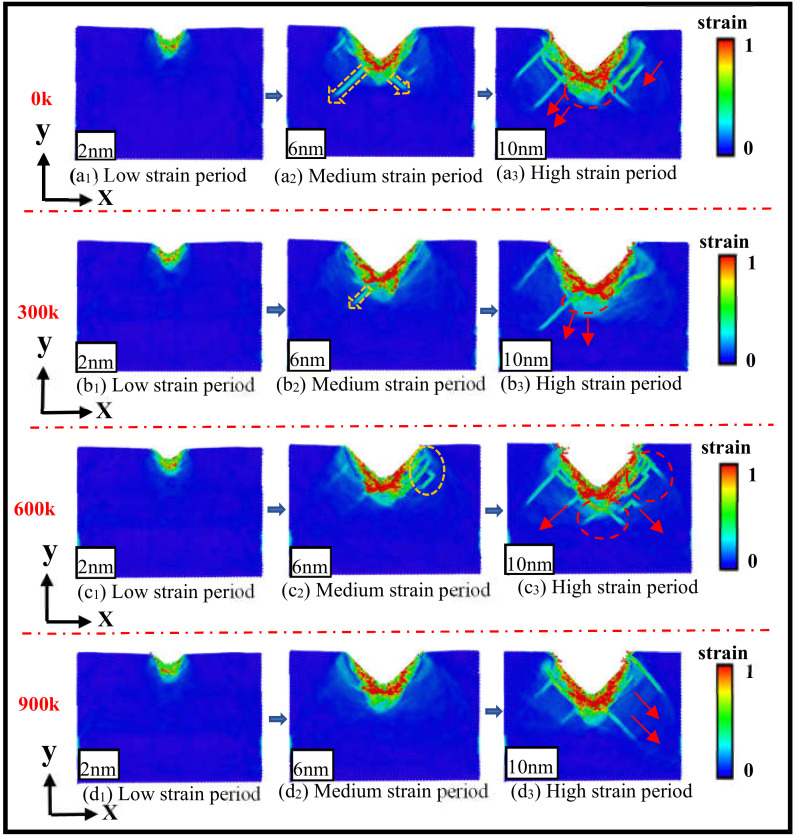
Deformation comparison analysis of different temperature indentation processes.

In this paper, the strain state of three-dimensional silicon carbide materials during nanoindentation loading at different temperatures was studied, and the damage of 3C-SiC under different temperatures was investigated. As shown in Figure 3, it is a strain cloud map of different temperatures and different indentation processes in the x0y-plane; the strain region corresponding to the color from blue to red is from the high strain zone to the low strain region, and the nanoindentation simulation temperatures 0 K, 300 K, 600 K and 900 K correspond to the Figure 3a–d, respectively. When the depth of the nanoindentation is 2 nm, compared with the graphs (a1), (b1), (c1) and (d1), the depth strain is not significantly different at different temperatures, and only shows low strain, and the degree of deformation of the matrix is small under this indentation depth. In Figure 3a2–d2, when the indentation depth is 6 nm, the contact area between the indenter and the 3C-SiC matrix shows a high strain zone with a different area due to the different temperature. The area of the high strain area is the largest at 600 K, indicating that the indentation surface deformation degree is the most significant at medium and high temperatures. The second area is from large to small 900 K, 300 K, 0 K. At 900 K, the strain is distributed in a clustered state; the stress action at 0 K, 300 K and 600 K is different; the strain spreads or expands in different directions and degrees. At 0 K, a large number of atoms slip in the negative direction of the x0y-plane, resulting in a long dislocation. At 300 K, 3C-SiC is stressed to reach the critical strength of dislocation germination. At the initial expansion trend at 600 K, in the positive direction of the x0y-plane, active ring dislocation extends downward along the negative direction of the *y*-axis. When the indentation depth reaches a maximum depth of 10 nm, the high strain zone expands in different ways at different temperatures compared to the comparison plots (a3), (b3), (c3) and (d3). At 0 K, three longer dislocations are derived at the mirror image in the negative direction of the x0y-plane. The extension trend is obvious and continuous. The 300 K and 600 K high strain regions are expanded with filler and point-fill limitations, respectively. The 300 K original dislocation continues to grow in the original direction while deriving new dislocations along 1/2[−110]; the 600 K strain is significantly extended by a different stress range and strength, but not continuous; the 900 K stress concentration, high strain region expansion is significant, but the degree of ductility is not significant, and the impact on internal damage is small. In summary, temperature has an effect on the damage state of the 3C-SiC matrix during nanoindentation loading. At different temperatures, there are differences in the strength, expansion direction and type of stress; the external characterization of strain is centered on the indenter contact area, and the tendency of extension occurs in different ways. Continuous and obvious extension occurs at a low temperatures; three long dislocations are derived in the negative direction and mirror image of the x0y-plane; a small number of ring dislocations and short dislocations are generated at the bottom of the indentation. The medium and high temperature strain is extended in a dispersed short section; there are cross-dislocations, discrete dislocations and centralized ring dislocations at the same time; compared with the limited effect of normal-temperature and high-temperature stress, the strain extension trend is not obvious; low, medium and high temperature have a significant impact on the internal damage of the matrix.

### 4.3. Dislocation Evolution and Extension Analysis

**Figure 4:**
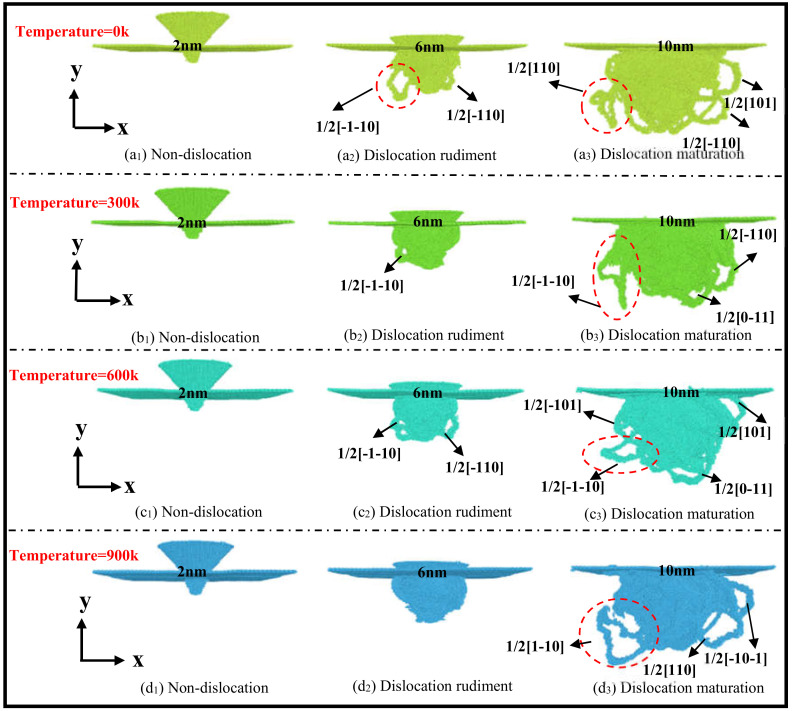
Flowchart of dislocation evolution and expansion.

Dislocation formation is accompanied by the generation of dislocation formation energy, resulting in an increase in the energy of the system; under the continuous joint action of various forces and stresses, atoms move, and crystals slip along different Burgers vectors to varying degrees, marking dislocation nuclei, dislocation value-added and motion [22]. As shown in Figure 4, the flowchart of dislocation evolution and expansion in indentation loading at different temperatures of (010) crystal planes, the yellow, green, cyan and blue dislocations correspond to the dislocation evolution and expansion at 0 K, 300 K, 600 K and 900 K temperatures, respectively. When the indentation depth is 2 nm, the comparison plot (a1), (b1), (c1) and (d1) shows that the 3C-SiC matrix does not produce dislocations at four temperatures; the interatomic covalent bond binding is stable, and there is no plastic damage on the surface of the matrix. When the indentation depth is 6 nm, it can be seen from the Figure 4a2–d2 that the system energy is relatively active at low and medium temperatures, while, at room temperature and high temperature, the static zone is significant. Among them, a long dislocation ring is formed at 1/2[−1−10] at 0 K; the covalent bond between atoms is broken; the energy is released; the deformation occurs inside the crystal; at the same time, a dislocation nucleus emerges at 1/2[−110], and the stress focus tends to act in the direction of 1/2[−110]. At 600 K, three dislocation nuclei appear at 1/2[−1−10] and [−110], while at 300 K only one dislocation nucleus appears at 1/2[−1−10]; there is no significant slip in the crystal plane; the system energy remains relatively stable. At 900 K, the energy is relatively stable. The dislocation ring or dislocation nucleus has not yet been generated. The number of covalent bonds between atoms is small. The degree of matrix damage is relatively small. When the indentation is the maximum depth of 10 nm, the comparison plot (a3), (b3), (c3) and (d3) shows that at 0 K, the atomic movement velocity is parallel to the slip line; a downward dislocation is generated along 1/2[110], releasing energy; at the same time, at the 1/2[−110] and 1/2[101] crystal orientation, two longer dislocation loops with horizontal expansion tendencies are generated, and plastic damage occurs on the substrate. At 300 K, the dislocation nucleus at 1/2[−1−10] evolves into a vertical downward suspension dislocation; the atomic velocity is perpendicular to the slip line; the dislocation climbs occurred; at the same time, two dislocation rings extending in the suspension direction are generated at 1/2[0−11] and 1/2[−110], resulting in certain deformation. At 600 K, the dislocation nucleus at 1/2[−1−10] evolves into a horizontal long dislocation ring; the covalent bond breaks in large quantities, releasing the dislocation formation energy, so that the elastic deformation is converted into a plastic deformation. At 900 K, a long dislocation with a vertical upward expansion trend is generated at 1/2[1−10]; two long dislocations are generated at 1/2[110] and 1/2[−10−1]; the dislocation value-added rate is significantly faster than the dislocation value-added rate at low, room and high temperatures.

**Figure 5:**
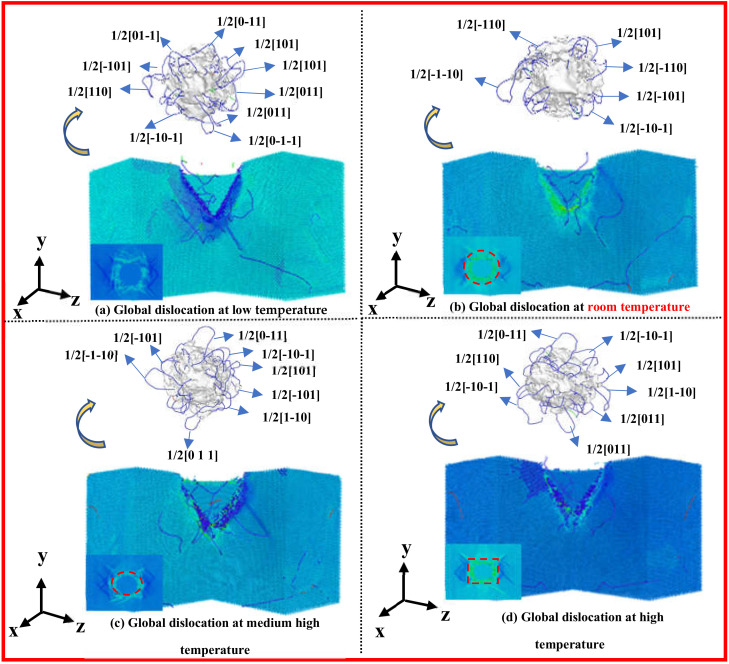
Dislocation panoramic view and stress-strain cross-section and overhead blueprint.

More clearly and accurately analyzing the evolution and expansion of dislocations at different temperatures, Figure 5 shows the dislocation panorama view and stress strain profile and overhead blueprint formed inside the single crystal silicon carbide at 0 K, 300 K, 600 K and 900 K temperatures at an indentation depth of 10 nm. Figure 5a–d, respectively, represent dislocation evolution at temperatures of 0 K, 300 K, 600 K and 900 K. From the stress strain cross-sectional blueprint, it can be seen that the high strain regions at each temperature are concentrated in the area in contact with the indenter, but the dislocation evolution and expansion are significantly different from the temperature effects. At 0 K, the long dislocation is more concentrated in the negative direction of the x0y-plane by the dislocation panoramic view; the stress spreads laterally along the x axis in the diagram, and the strain region also expands along the x axis. At 300 K, the dislocations generated have the characteristics of a short length and concentrated distribution on both sides; at the same time, it is found that the stress acts in a divergent radiation around it; the strain area is basically circular. At 600 K, a small number of longer dislocations generated are concentrated in the x0z-plane; most of the short dislocations are generated in the x0y-plane; and the distribution of the high strain region has a point-like trend. The stress also shows lateral diffusion; the strain region is elliptical. At 900 K, the generated dislocations have the characteristics of a long length and four-sided unfolding distribution; stress concentration is found, and the strain zone diffuses in the form of moments. In summary, temperature has an impact on the evolution and expansion of dislocations in 3C-SiC crystals during nanoindentation loading; macroscopic characterization has differences in the crystal damage degree and state at different temperatures. Under low-temperature conditions, various forces and stress dispersions work together; there is a spiral dislocation of the slip movement, a vertical climbing dislocation at room temperature, indicating that the energy and force conditions are different. There is a horizontal long dislocation produced at medium and high temperatures, most of which are mainly short dislocations; the energy distribution is uneven; the high strain is the dotted distribution; the dislocation value-added rate is fast at high temperatures; and the energy change rate is high. Different degrees of plastic damage occurred inside the matrix, which proves that different temperatures have an effect on the evolution and expansion of dislocations during the loading of nanoindentation of the 3C-SiC matrix.

### 4.4. Potential Energy Change and Load Displacement Curve Analysis

**Figure 6:**
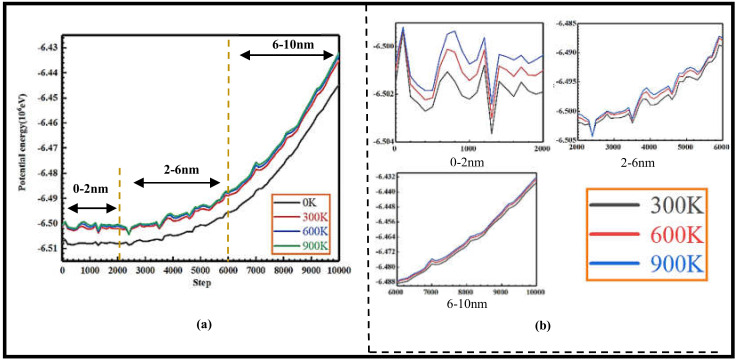
Graph of potential energy-step change at different temperatures. (**a**) Potential energy step relationship at low, normal medium high and high temperature, (**b**) Potential energy step relationship at normal, medium high and high temperature.

The system potential energy change corresponds to the damage and expansion energy change of the 3C-SiC crystal in the indentation loading; as shown in Figure 6, it is the potential energy-step change curve at 0 K, 300 K, 600 K and 900 K temperatures; as can be seen from Figure 6a, the potential energy-step curve under the four temperature conditions are gradually rising with the depth of the downward pressure, indicating that the system potential energy during the loading process is a reduced change. At 900 K, the potential energy is reduced the most; the potential energy value at 600 K and 300 K is reduced in turn, but the change in high temperature, medium high temperature and room temperature is slightly different; at 0 K, the potential energy value rises with the depth of the downward pressure, but the degree of reduction is significantly lower than the degree of potential energy reduction at normal temperature, medium-high temperature and high temperature. As shown in Figure 6b, the specific analysis of the potential energy change under various temperature conditions, respectively, compared with 0–2 nm, 2–6 nm and 6–10 nm depths of the potential energy value change difference, can be seen, from the microscopic point of view, due to the increase in temperature; the distance between the atoms of the specimen will become larger, the attraction and repulsion between the atoms will be weakened; the potential energy will be reduced.

**Figure 7:**
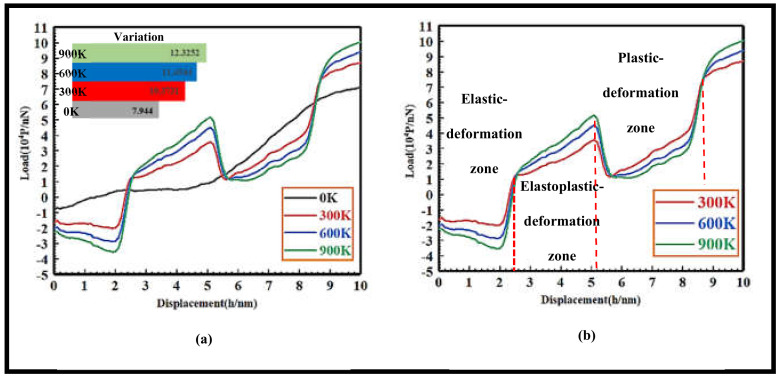
Comparison of load-displacement curves at different temperatures. (**a**) Low, normal, medium and high temperature load displacement curve, (**b**) Normal, medium and high temperature load displacement curve.

As shown in Figure 7, it is a load-isplacement curve when indentation is loaded at different temperatures; as shown in Figure 7a is a load-displacement curve at low, room, medium-high and high temperature; the initial pressure of the indenter is the highest 0 K download load value, followed by 300 K and 600 K load values; the 900 K download load value is the lowest. The change in the load displacement curve at 300 K, 600 K and 900 K temperatures is basically synchronized; the load value changes with the displacement from large to small to 900 K > 600 K > 300 K > 0 K; there is a significant elevation at 2.5 nm and 5.2 nm, but the load displacement curve changes relatively gently at 0 K, and there is no significant elevation. As can be seen in Figure 7b, the download load values at 300 K, 600 K and 900 K can be divided into four regions with the change in indentation displacement: elastic, elastoplastic, plastic and complete plastic deformation area. When the indentation displacement is 0–2.5 nm, the atomic lattice in the contact area between the indenter and the matrix undergoes a recoverable elastic deformation, which is the elastic deformation region. At 2.5–5.2 nm, the stress increases to a specific value; the strain energy stored in the atomic lattice begins to be released; the degree of deformation begins to transition from the elastic deformation stage to the plastic deformation stage, which is the elastoplastic deformation region. At 5.2–8.8 nm, a large amount of strain energy is released; the atomic lattice is seriously deformed; dislocations are generated inside the crystal, which is a plastic deformation region. At 9.2–10 nm, the internal extrusion of the matrix undergoes a serious irreversible complete plastic deformation, resulting in a large number of dislocations, which is highly consistent with the dislocation evolution and expansion process in the loading process of the 3C-SiC indentation at different temperatures, which further tests the accuracy of the test.

## 5. Conclusions

(1) The influence of the degree of interatomic aggregation, radial distribution function and interatomic force on the 3C-SiC nanoindentation loading process is not obvious due to the different temperature environments of the system. This reflects the good heat resistance of single crystal SiC material to some extent. The damage degree of low and medium-high temperature on single crystal 3C-SiC is higher than that at room temperature and high temperature. In addition, the stress intensity, direction and type of extension are affected by temperature, and the strain extension range, direction and aggregation discrete type have obvious differences.

(2) Compared with previous studies, it was found that temperature has a great influence on the microdislocation of single crystal 3C-SiC. The dislocation slip at low temperature, dislocation climbing at room temperature, dislocation ring expansion at middle and high temperature and dislocation growth rate of high temperature are closely related to the effect of temperature on the behavior of atomic lattice change during the test loading process. The difference in dislocation generation, movement and increment behavior at different temperatures corresponds to the representation of the load displacement function in the same environment. It has certain guiding significance to the temperature environment of single crystal 3C-SiC processing and application.

## Figures and Tables

**Table 1:** Model parameters of 3C-SiC nanoindentation.

The Relevant Parameter	Parameter Values
Simulated box size Number of atoms in simulated box	24 nm × 27.5 nm × 24 nm 1,039,657
Indenter diameter Height of the indenter Number of atoms in cone head	8 nm 5.3 nm 24,409
3C-SiC Workpiece size	24 nm × 17 nm × 24 nm
Indentation crystal Ensemble Pushing speed Depth of indentation Temperature Timestep	(010) (NPT) 20 m/s 10 nm 0 K, 300 K, 600 K, 900 K 1 fs

## Data Availability

Not applicable.
